# Serum Trace Elements and Their Associations with Breast Cancer Subgroups in Korean Breast Cancer Patients

**DOI:** 10.3390/nu11010037

**Published:** 2018-12-24

**Authors:** Rihwa Choi, Min-Ji Kim, Insuk Sohn, Serim Kim, Isaac Kim, Jai Min Ryu, Hee Jun Choi, Jae-Myung Kim, Se Kyung Lee, Jonghan Yu, Seok Won Kim, Seok Jin Nam, Jeong Eon Lee, Soo-Youn Lee

**Affiliations:** 1 Department of Laboratory Medicine and Genetics, Samsung Medical Center, Sungkyunkwan University School of Medicine, 81 Irwon-ro, Gangnam-gu, Seoul 06351, Korea; pirate0720@naver.com; 2 Department of Laboratory Medicine, Green Cross Laboratories, Gyeonggi, Yongin 16924, Korea; serim@gclabs.co.kr; 3 Statistics and Data Center, Research Institute for Future Medicine, Samsung Medical Center, Seoul 06351, Korea; rabbit93.kim@samsung.com (M.-J.K.); insuk.sohn@samsung.com (I.S.); 4 Breast Division, Department of Surgery, Samsung Medical Center, Sungkyunkwan University School of Medicine, 81 Irwon-ro, Gangnam-gu, Seoul 06351, Korea; 24icecream@hanmail.net (I.K.); snowman120@hanmail.net (J.M.R.); heejun1.choi@samsung.com (H.J.C.); jmjidia@hanmail.net (J.-M.K.); sekyung.lee@samsung.com (S.K.L.); lymbics@hanmail.net (J.Y.); seokwon1.kim@samsung.com (S.W.K.); seokjin.nam@samsung.com (S.J.N.)

**Keywords:** breast cancer, trace elements, molybdenum, manganese

## Abstract

The relationships between serum levels of trace elements and breast cancer remain relatively unknown. In this study, we investigate serum levels of seven trace elements in Korean breast cancer patients compared to controls without breast cancer. Serum trace element levels were determined using inductively coupled plasma mass spectrometry in Korean breast cancer patients before initiation of breast cancer treatment. Korean females without breast cancer served as a control group. Trace element levels were measured in the discovery cohort (*n* = 287) and were validated in an independent cohort (*n* = 142). We further investigated possible associations between trace element levels and the presence of lymph node metastasis, distant metastasis, or triple-negative breast cancer among breast cancer patients in subgroup analyses. Serum manganese and molybdenum levels were significantly higher (*p* < 0.05) in breast cancer patients than in controls. Serum copper levels were significantly higher in breast cancer patients with distant metastasis, while selenium levels were significantly lower. Other trace elements were neither significantly different between breast cancer patients and controls nor between subgroups of breast cancer patients. Our study provides insights about the potential roles and impacts of trace elements through an assessment of the associations between trace elements and breast cancer.

## 1. Introduction

Every year, 1.7 million women are diagnosed with breast cancer, making it the most common cancer in women worldwide and with an estimated 522,000 deaths in 2012, the leading cause of cancer death in women (accounting for 15% of all cancer deaths) [1]. A comparative analysis of epidemiological data for breast cancer in the USA, Canada, India, China, Taiwan, Japan, South Korea, and Sweden showed notable differences in the median age at diagnosis; the peak age at diagnosis is 40–50 years in Asian countries, and 60–70 years for Western countries [1,2,3]. Increasing age, family history of breast cancer, major inheritance susceptibility, breast density, hormone-based therapies, exposure to ionizing radiation, obesity and alcohol consumption are major associated risks associated with breast tumors [4].

Although many studies have focused on breast cancer etiology, nosogenesis and treatment, aspects of this disease remain poorly defined [5]. A comprehensive understanding of trace element speciation, localization and function under various pathophysiological conditions is becoming increasingly important for understanding disease mechanisms and discovering novel diagnostic, prognostic, and therapeutic targets for breast cancer [6]. Little is known about beneficial or harmful relationships between serum trace element levels and breast cancer [4]. Trace elements play important roles in biological processes relevant to breast cancer, especially those elements that are essential components of antioxidants [5]. Certain environmental and nutritional factors may elicit estrogenic and/or carcinogenic effects, influencing breast cancer [4].

Although some researchers have worked to identify associations between trace element levels and breast cancer in different populations using samples such as hair, toenails, or blood (including serum and plasma), their results have been inconsistent. Different analytical methods can be used to identify and measure trace elements, such as X-ray fluorescence spectroscopy, atomic absorption spectroscopy (AAS), inductively coupled plasma mass spectrometry (ICP-MS), and inductively coupled plasma atomic emission spectroscopy (ICP-AES) [7]. Different assays vary in sensitivity and specificity for trace element determination, which may affect study results [8]. Most studies of trace element status in breast cancer that have been performed in humans included small numbers of subjects, limited numbers of trace elements, or were performed as long ago as the 1990s [9]. Furthermore, no reliable data have been collected regarding multiple serum trace element levels in Koreans, either from breast cancer patients or control females.

Therefore, in this study, we simultaneously measured serum levels of seven trace elements in Korean female breast cancer patients and compared these values with Korean female controls without breast cancer using ICP-MS. Furthermore, we investigated clinical and laboratory variables related to trace element levels in Korean breast cancer patients, as well as the possible associations between trace element levels and the presence of lymph node metastasis, distant metastasis, or triple-negative breast cancer among breast cancer patients.

## 2. Materials and Methods

### 2.1. Study Population

To evaluate whether trace element levels are factors in breast cancer risk among patients compared to controls, we established a discovery cohort consisting of 150 female breast cancer patients and 137 controls. We then assessed serum trace element levels in an independent validation cohort consisting of 79 breast cancer patients and 63 controls. For the discovery cohort, we enrolled 150 adult female patients with histopathological diagnoses of breast cancer and 137 control subjects without breast cancer at Samsung Medical Center (Seoul, Korea), a tertiary care hospital, between September 2008 and April 2015. A control group of 137 female subjects without breast cancer was recruited from individuals who visited a health promotion center for medical checkups without any clinical symptoms or signs of breast cancer. For the validation cohort, 79 breast cancer patients and 63 controls (19 healthy female subjects and 44 females with benign breast disease, such as fibrocystic changes or fibroadenoma) were prospectively enrolled. Participants were recruited consecutively between May 2017 and October 2018. The exclusion criteria included diagnoses of any malignancy other than primary breast cancer. The overall study design is shown in Figure S1. All participants were adults (≥18 years of age). Demographic data including age and body mass index (BMI) were obtained from electronic medical records. Post-surgical information about breast cancer cells tested for estrogen receptors (ER), progesterone receptors (PR), and human epidermal growth factor receptor 2 (HER2) from breast cancer patients was obtained through electronic medical records; triple-negative cancer tested negative for all three of those markers. Further information about the presence of lymph node metastasis and distant metastasis were also obtained from electronic medical records for subgroup analyses.

### 2.2. Ethical Approval and Informed Consent

The study was conducted in accordance with the Declaration of Helsinki, and the protocol was approved by the Institutional Review Board of Samsung Medical Center (IRB No: SMC-2016-07-129). All subjects gave informed consent for inclusion before they participated in the study.

### 2.3. Analytical Procedures

Blood samples for determining trace element levels were collected from both cases and controls in the fasting state during the first visit. Serum samples were drawn for analysis before the initiation of cancer treatment in breast cancer patients in both discovery and validation cohorts. Each serum sample was immediately stored at −20 °C until the moment of analysis. Serum trace element concentrations were determined by ICP-MS (Agilent 7900, Agilent Technologies, Santa Clara, CA, USA). Multi-element determination was performed on an Agilent 7900 ICP-MS equipped with octopole reaction system collision/reaction cell technology to minimize spectral interference. Details regarding sample preparation and analytical methods are summarized in Material S1. The assay ranges were 0.00–80.16 µg/L for cobalt (Co), 0.00–78.98 µg/L for chromium (Cr), 0.0–406.0 µg/dL for copper (Cu), 0.00–80.92 µg/L for manganese (Mn), 0.0–81.0 µg/L molybdenum (Mo), 0.0–480.0 µg/L for selenium (Se), and 0.0–404.0 µg/dL for zinc (Zn). Intra-assay coefficients of variation (within-run precision) and inter-assay coefficients of variation (between-run precision) for all assays were <10%. Calibration curves were established (*r* > 0.999). The accuracy of serum trace element measures was assured using Quebec Multielement External Quality Assessment Scheme (QMEQAS) and the Proficiency Testing/Quality Management program of the College of American Pathologists (CAP) survey (United States). To assess biochemical status, serum chemistry parameters and lipid profiles including total protein, albumin, aspartate aminotransferase, alanine aminotransferase, alkaline phosphatase, total cholesterol, high-density lipoprotein (HDL), and low-density lipoprotein (LDL) were determined using a Roche modular analyzer (Roche Diagnostics Corp., Indianapolis, IN, USA).

### 2.4. Statistical Analysis

We used nonparametric methods (Wilcoxon rank sum test) when the data were not normally distributed. Data normality was assessed by the Shapiro–Wilk’s test. We used correlation analysis for continuous variables, *t*-tests and analysis of variance for categorical and continuous variables, and chi-square tests for categorical variables. *p*-values less than 0.05 were considered to be significant. Statistical analyses were executed using SAS version 9.4 (SAS Institute, Cary, NC, USA). 

## 3. Results

### 3.1. General Characteristics of the Study Population

In total, 287 Korean female subjects participated in the discovery cohort of this study: 137 controls without any breast lesions and 150 breast cancer patients. The median (interquartile range) ages were 48.0 years (43.0–56.0 years) for controls and 46.0 years (40.0–53.0) for patients, respectively. Among the 150 breast cancer patients, 84 (56.0%) had lymph node metastasis, 14 (9.3%) had distant metastasis, and 24 (16.0%) had triple negative breast cancer. In total, 142 Korean female subjects were included in the validation cohort: 63 controls (without breast cancer) and 79 breast cancer patients. The median (interquartile range) age was 41.0 years (31.0–48.0 years) for controls and 47.0 years (43.0–53.8) for patients, respectively. Among the 79 breast cancer patients, 11 (13.9%) exhibited lymph node metastasis, none (0.0%) had distant metastasis, and five (6.3%) had triple-negative breast cancer. The baseline characteristics of the study sample are summarized in Table 1 and Tables S1 and S2. BMIs were slightly higher in breast cancer patients than in controls. In the discovery cohort, data for determining serum total protein in both controls and cancer patients, age, serum total cholesterol, and LDL in controls, and Mn and Zn in cancer patients were normally distributed. In the validation cohort, data for age, BMIs, HDL, and Cu in both controls and cancer patients, serum total protein, albumin, and Cr in controls were normally distributed. Other data were not normally distributed.

### 3.2. Serum Trace Element Concentrations in Korean Breast Cancer Patients and Controls without Breast Cancer 

Median (interquartile range) serum trace element concentrations in breast cancer patients and controls without breast cancers are summarized in Table 1 and Tables S1 and S2. Among the seven trace elements, serum Mn and Mo were significantly different between breast cancer patients and controls (*p* < 0.05) in both the discovery and validation cohorts, while serum Co, Cr, Cu, Se, and Zn were not. Serum Mn and Mo were significantly higher in breast cancer patients than in controls (*p* < 0.05). Among the seven trace elements, there were weak positive correlations between Co and Mn (*ρ* = 0.1197 and *p* = 0.0427 in the discovery cohort; *ρ* = 0.2210 and *p* = 0.0081 in the validation cohort), and Se and Cu (*ρ* = 0.2216 and *p* = 0.0002 in the discovery cohort; *ρ* = 0.3860 and *p* = < 0.0001 in the validation cohort, Table S3) in both the discovery and validation cohorts.

### 3.3. Subgroup Analysis—Serum Trace Element Concentrations in Breast Cancer Patients

**Figure 1:**
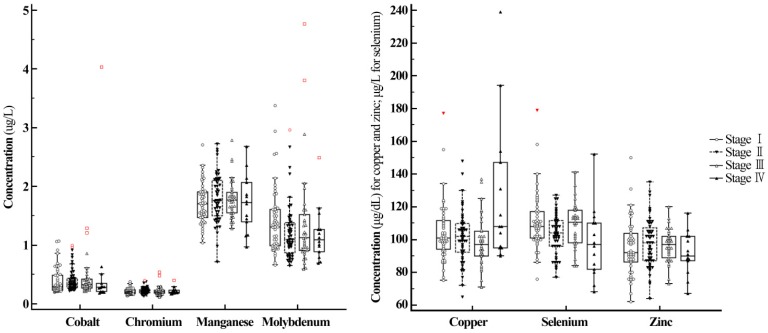
Serum levels of seven trace elements among breast cancer patients at different stages. Among seven trace elements, concentrations of Cu were significantly higher and concentrations of Se were significantly lower in patients with stage IV breast cancer (metastatic breast cancer) than in patients at other breast cancer stages (stage I to III breast cancer).

Concentrations of the seven trace elements were compared between breast cancer patients with and without lymph node metastasis, and no significant differences were found in the discovery cohort (Table 2). In the discovery cohort, among seven trace elements, data for Mn and Zn in patients with and without lymph node metastasis were normally distributed and other data were not. In the validation cohort, data for Cu and Zn in both patients with and without lymph node metastasis and Co and Se in patients with lymph node metastasis were normally distributed. Other data were not normally distributed. Concentrations of the seven trace elements were compared among breast cancer patients in different stages. No significant differences were observed in seven trace element levels among breast cancer patients at different stages. However, among the seven trace elements, concentrations of Cu were significantly higher and concentrations of Se were significantly lower in women with stage IV breast cancer in the discovery cohort (metastatic breast cancer, Figure 1 and Table 2). In the discovery cohort, among seven trace elements, data for Mn and Zn in patients with and without stage IV breast cancer were normally distributed and data for the others were not. No breast cancer patients with stage IV breast cancer were enrolled in the validation cohort.

Concentrations of the seven trace elements were compared between breast cancer patients with and without triple-negative breast cancer. In the discovery cohort, data for Mn in patients with and without triple-negative breast cancer were normally distributed. Data for Zn in patients without triple-negative breast cancer and Se in patients with triple-negative breast cancer were normally distributed in the discovery cohort. The nonparametric method for comparison of trace element concentrations except for Mn was used in the discovery cohort. In the validation cohort, data of the seven trace elements were not normally distributed in patients without triple-negative breast cancer, and the nonparametric method for comparison was used. No significant differences were found in the concentrations of the seven trace elements between the two groups in either the discovery or validation cohorts (see Table 2 for the discovery cohort; data not shown for the validation cohort).

## 4. Discussion

In this study, we simultaneously measured serum levels of seven trace elements using ICP-MS in Korean female breast cancer patients and controls without breast cancer. We also investigated possible associations between trace element levels and the presence of lymph node metastasis, distant metastasis, or triple-negative breast cancer among breast cancer patients. 

Numerous studies have suggested that certain environmental compounds such as phytoestrogens, xenoestrogens and metalloestrogens exhibit estrogen-like properties, including the ability to influence breast carcinogenesis [4]. However, trace elements are also essential components of antioxidants and may exert protective effects as well [5]. In this study, serum Mn and Mo levels were significantly higher (*p* < 0.05) in breast cancer patients than in controls. The relationship between trace elements and cancer etiology and progression has been extensively studied, with contradictory results [5]. Previous studies of trace element concentrations performed in different geographic regions using different specimens are summarized in Table 3. There have been inconsistencies regarding trace element status in breast cancers among studies. Previous studies of trace elements using whole blood, serum, and plasma samples in breast cancer patients compared with controls are summarized in Table 4. In the present study, Co levels were significantly higher in breast cancer patients than in controls in the discovery cohort, results that are comparable to the findings of previous studies performed in China [10]. Co levels were not significantly different between the two groups in the validation cohort, a result that is comparable to the findings of previous studies performed in Taiwan [11] and Nigeria [12], which reported no significant differences between the two groups. For Cr, although levels were higher in breast cancer patients in the validation cohort, comparable to the results of a previous study performed in Taiwan [11], no significant differences between the two groups were observed in our discovery cohort or in two other studies performed in China [10] and Canada [13]. However, the Canadian study included breast cancer patients with the *BRCA1* mutation [13]. Several studies performed in China [10,14], Taiwan [11], India [15], and Nigeria [12,16] reported higher levels of Cu in breast cancer patients than in controls, while the opposite finding (significantly higher Cu levels in controls than in breast cancers) was reported in Kuwait [17], and no difference was found in a study performed in Canada [13] or in India [18]. For Mn, although significantly higher levels were observed in breast cancer patients in this study, other studies performed in China [10,14] found significantly lower levels in breast cancer patients. Moreover, the range of Mn concentrations in the Korean subjects observed in this study were lower than in the Chinese studies. For Se and Zn, significantly lower concentrations were observed in breast cancer patients than in controls in the discovery cohort of this study, which was comparable to the results of previous studies performed in Nigeria [16], China [14], and Kuwait [17]. However, no significant differences in concentrations of Se were observed in the validation cohort in this study, similar to the results of a study performed in Canada [13] and another performed in China [10]. A recent systematic review reported that serum Se concentrations were correlated with breast cancer and could be used to predict the disease [9]. Another updated meta-analysis and meta-regression study reported that Se exposure decreased the risk of breast cancer [19].

Differences in trace element concentrations among different populations could reflect geographical location, cultural practices, pollution, or differences in body composition and genetics [30]. Genetic loci affecting blood Cu, Se, and Zn levels in Australian and British samples were identified through a recent genome-wide association study [31]. 

A recent meta-analysis of 14 previous studies reported no significant differences in serum Zn levels between female breast cancer patients and control subjects, but also described considerable heterogeneity among the included studies (*I*^2^ 90.2% to 97.8%) [5]. Differences in study design, number of subjects, characteristics of breast cancer patients (including specific subtypes of breast cancers with histopathological and molecular/genetical differences), sampling time (pre- or post- treatment), or analytical methods (X-ray fluorescence spectroscopy, AAS, ICP-MS, etc.) could also impact the heterogeneity of results [32].

Axillary lymph node status is one of the most important prognostic factors in patients with primary breast cancer and provides critical information to guide treatment decisions [32,33]. Currently, decisions regarding adjuvant therapy are based largely on primary cancer characteristics such as tumor grade and ER and HER2 status, as well as other molecular features, rather than the number of positive lymph nodes [34]. The St. Gallen 2013 Consensus Guidelines Panel stated that the effectiveness of chemotherapy did not depend on the number of positive nodes, but on the underlying tumor biology [34,35]. Although several studies have compared trace element concentration in cancerous and benign breast tumor tissues and/or normal breast tissues [12,18,20,36], the impact on tumor biology and etiology during lymph node involvement in early breast cancer remains poorly defined. Previous studies have focused on comparing normal and malignant tissues. For example, Se has been shown to strengthen tight junctions and reinforce cell-cell attachment in breast cancer, which is consistent with the antiproliferative effects of Se [37]. Blood or serum Se levels are reportedly lower in breast cancer patients, while tissue Se concentration is higher in malignant tissues (representing an inverse correlation) [20,38]. In this study, although we did not measure tissue concentrations of trace elements, we compared serum trace element concentrations in breast cancer patients with and without lymph node metastasis to focus on possible associations between trace elements and lymph node involvement in early breast cancer. Although no associations were observed among the seven trace elements tested between the two groups, future studies are needed with large numbers of samples that include more tissue types as well as lymph nodes.

Next to prolongation of survival, the primary therapeutic goals in metastatic breast cancer are maintenance of quality of life and palliation of symptoms [32]. Therapeutic concepts are determined by a multidisciplinary team at the initiation of treatment, and different therapeutic steps with different regimens are needed for different breast cancer patients [32]. In this study, Cu levels were significantly higher in women with stage IV breast cancer (metastatic breast cancer) than in women without stage IV breast cancer and Se levels were significantly lower in women with stage IV breast cancer than in those without stage IV breast cancer (*p* < 0.05). Since Cu is a key component in many essential enzymes, Cu is required for at least three characteristic phenomena involved in cancer progression: Proliferative immortality, angiogenesis, and metastasis [39]. Meanwhile, Se has been shown to strengthen tight junctions and reinforce cell-cell attachment in breast cancer [37] and in vitro evidence suggests that Se impacts both endothelial and cancer cells, leading to the reduction of major regulatory molecules in angiogenesis [37]. Accordingly, Cu and Se represent possible targets for diagnosis, prognosis and management in breast cancer [40]. However, there is insufficient evidence to provide clinician and patient guidelines on the use of antioxidant supplements including Se during breast cancer treatment [41]. Due to the potential heterogeneity between metastases, differences in treatment regimens could also affect trace element status in metastatic breast cancer patients [32]. Well-designed future studies are needed to determine the significance of trace elements in metastatic breast cancer patients.

Triple-negative breast cancer presents clinical challenges due to unknown etiology, lack of treatment targets, and poor prognosis [29,42]. Chemotherapy is always indicated in triple-negative breast cancer after endocrine options have been exhausted in luminal disease or if rapid response is needed in life-threatening situations or in patients who are highly symptomatic [32]. Although in vitro and in vivo studies of normal and triple-negative breast cancer cells have assessed whether a combination of cisplatin and tumor necrosis factor-related apoptosis-inducing ligand (TRAIL) can improve therapeutic outcomes [43], there is a lack of studies exploring serum trace element status in triple-negative breast cancer patients. Multiple genetic and nutritional factors were associated with triple-negative breast cancer in a study of Canadian women [42]. However, that study only focused on dietary intake, finding that dietary intake of Zn was associated with triple-negative breast cancer [42]. Although we observed no significant differences in trace element concentrations between breast cancer patients with and without triple-negative breast cancer in the current study, our sample size of triple-negative breast cancer patients was small. Future studies with large numbers of in different ethnic populations are needed to elucidate associations between trace element status and triple-negative breast cancer.

The strengths of our study are the use of an accurate ICP-MS method to measure multiple trace elements simultaneously in a relatively large cohort of Korean breast cancer patients and controls and its prospective design. Furthermore, we assessed the relevance of serum trace element concentrations in lymph node metastasis, stage IV breast cancer, and triple-negative breast cancer, which have received little attention before. This study provides background information to support further explorations of trace elements in breast cancer etiology. Future studies to assess relationships among serum concentration, tissue concentration, nutrient supplementation, and treatment outcomes are needed to improve breast cancer patient care. 

Our study has several limitations. Although we assessed several biochemical factors, information about dietary intake was not evaluated in our investigation. We also did not measure ceruloplasmin (the major Cu-carrying protein) or other proteins involved in antioxidant pathways (glutathione peroxidase enzymes and selenoprotein P, etc.), which might alter trace element concentrations [14]. Differences in study population characteristics between the discovery and validation cohorts could have affected the final results. Such differences could explain the inconsistencies in the associations between Co, Cr, Cu, and Zn and breast cancer between the discovery cohort and the validation cohort observed in this study. Stage IV breast cancer patients were not enrolled in the prospectively designed validation cohort. Future studies considering these factors are needed to clarify the clinical significance of changes in trace element concentrations.

## 5. Conclusions

In conclusion, we investigated the serum levels of multiple trace elements in Korean breast cancer patients and control subjects for the first time. Serum concentrations of Mn, and Mo were significantly higher in breast cancer patients than in controls (*p* < 0.05). Among breast cancer patients, serum concentrations of Cu were significantly higher in patients with stage IV breast cancer, while concentrations of Se were significantly lower (*p* < 0.05). Our study provides insights regarding the potential roles and impacts of trace elements in breast cancer.

## Figures and Tables

**Table 1:** Characteristics and levels of seven trace elements in study subjects and risk of breast cancer.

	1st Dataset for Discovery Cohort							2nd Dataset for Validation Cohort						
	Control			Breast Cancer			*p*-Value ^a^	Control		Breast Cancer				*p*-Value ^a^
	*n*	Median	IQR	*n*	Median	IQR		*n*	Median	IQR	*n*	Median	IQR	
Age (years)	137	48.00	42.75–56.00	150	46.00	40.00–53.00	0.1860	63	41.00	31.00–48.00	79	47.00	43.00–53.75	<0.0001
Body Mass Index (kg/m^2^)	137	21.40	19.80–23.80	150	22.54	20.80–24.53	0.0007	63	21.45	19.86–23.23	79	23.11	21.32–24.82	0.0008
Serum Total Protein (g/dL)	137	7.10	6.80–7.40	150	7.30	7.10–7.60	0.0001	63	7.30	7.00–7.50	79	7.40	7.10–7.70	0.0390
Serum Albumin (g/dL)	137	4.30	4.20–4.60	150	4.70	4.50–4.80	<0.0001	63	4.60	4.50–4.78	79	4.60	4.50–4.80	0.8262
Serum Total Cholesterol (mg/dL)	137	193.00	172.00–218.00	150	184.50	163.00–210.00	0.0331	63	169.00	157.50–192.50	79	177.00	163.00–202.75	0.1664
Aspartate Transaminase (IU/L)	137	20.00	17.00–24.00	150	18.00	15.00–21.00	0.0047	63	17.00	15.00–21.00	79	17.00	15.00–21.00	0.9311
Alanine Transaminase (IU/L)	137	15.00	11.00–20.25	150	14.00	11.00–21.00	0.3931	63	12.00	10.00–17.75	79	14.00	12.00–18.00	0.0436
Alkaline Phosphatase (U/L)	137	54.00	44.00–67.00	149	57.00	47.00–72.00	0.1220	63	52.00	43.00–67.75	79	53.00	45.25–64.00	0.6192
HDL (mg/dL)	129	62.00	53.00–72.25	75	58.00	50.00–67.00	0.1095	42	68.50	60.00–78.00	72	62.50	51.00–73.00	0.0181
LDL (mg/dL)	130	117.00	98.00–138.00	75	115.00	95.50–135.75	0.7899	42	102.50	93.00–124.00	72	109.00	95.00–135.00	0.2218
Cobalt (µg/L)	137	0.24	0.18–0.36	150	0.31	0.25–0.43	<0.0001	63	0.45	3.23–0.59	79	0.41	0.25–0.56	0.1787
Chromium (µg/L)	137	0.20	0.18–0.23	150	0.21	0.18–0.24	0.0767	63	0.18	0.15–0.23	79	0.27	0.20–0.31	<0.0001
Copper (µg/dL)	137	95.00	87.00–103.00	150	100.00	93.00–111.00	0.0002	63	92.00	81.25–102.50	79	90.00	82.00–96.75	0.2805
Manganese (µg/L)	137	1.54	1.04–1.71	150	1.75	1.49–2.00	<0.0001	63	0.58	0.53–0.68	79	0.64	0.56–0.78	0.0099
Molybdenum (µg/L)	137	1.05	0.88–1.30	150	1.16	0.95–1.50	0.0039	63	1.10	0.90–1.20	79	1.20	0.93–1.40	0.0056
Selenium (µg/L)	137	109.00	102.00–119.00	150	107.00	98.00–114.00	0.0241	63	102.00	95.00–112.00	79	98.00	92.00–112.50	0.0845
Zinc (µg/dL)	137	110.00	97.75–126.00	150	95.00	87.00–104.00	<0.0001	63	75.00	67.00–85.00	79	76.00	72.00–82.75	0.5961

Abbreviations: BMI, body mass index; ALP, alkaline phosphatase; HDL, high-density lipoproteins; IQR, interquartile range; LDL, low-density lipoprotein. ^a^ *p*-values based on the Wilcoxon rank sum test for nonparametric data and *t*-test for parametric data (protein in 1st dataset and age, BMI, HDL, and Copper in 2nd dataset were normally distributed.).

**Table 2:** Subgroup analyses for seven trace elements in 150 breast cancer patients in the discovery cohort.

	Lymph Node Metastasis					Stage IV Breast Cancer					Triple-Negative Cancer				
	No (*n* = 66)		Yes (*n* = 84)		*p*-Value	No (*n* = 136)		Yes (*n* = 14)		*p*-Value	No (*n* = 126)		Yes (*n* = 24)		*p*-Value
	Median	IQR	Median	IQR		Median	IQR	Median	IQR		Median	IQR	Median	IQR	
Cobalt (µg/L)	0.30	0.25–0.49	0.32	0.26–0.43	0.6699	0.31	0.26–0.44	0.29	0.21–0.35	0.1328	0.31	0.26–0.44	0.27	0.25–0.38	0.2463
Chromium (µg/L)	0.21	0.18–0.24	0.21	0.18–0.24	0.7977	0.21	0.18–0.24	0.20	0.18–0.23	0.5707	0.21	0.18–0.24	0.20	0.19–0.23	0.3862
Copper (µg/dL)	101.00	93.00–111.00	100.00	93.00–110.00	0.6908	100.00	92.00–110.00	108.00	95.00–147.00	0.0479 ^b^	100.50	93.00–110.00	99.00	92.50–112.00	0.8818
Manganese (µg/L)	1.72	1.49–2.01	1.75	1.49–1.99	0.8011 ^a^	1.75	1.50–1.99	1.73	1.40–2.07	0.6716 ^a^	1.76	1.49–2.02	1.63	1.48–1.83	0.2243 ^a^
Molybdenum (µg/L)	1.22	0.95–1.57	1.13	0.95–1.44	0.2899	1.18	0.96–1.50	1.10	0.89–1.27	0.4009	1.19	0.96–1.50	1.02	0.93–1.50	0.5183
Selenium (µg/L)	107.00	100.00–115.00	107.50	96.00–114.00	0.4133	108.00	99.00–114.50	97.00	82.00–110.00	0.0437 ^b^	107.50	99.00–114.00	103.50	97.50–119.00	0.8899
Zinc (µg/dL)	95.00	87.00–106.00	95.00	87.00–104.00	0.6801 ^a^	96.00	87.00–105.00	90.00	87.00–102.00	0.2190 ^a^	96.00	87.00–105.00	91.50	87.00–101.00	0.6517

IQR, interquartile range. *p*-values based on the Wilcoxon rank sum test for nonparametric data and ^a^ *t*-test for parametric data; ^b^ *p* < 0.05.

**Table 3:** Previous studies of trace elements in breast cancer patients compared to controls.

Region	*n* of Breast Cancer	*n* of Controls	Specimen	Tested Trace Elements	Significantly Low in Breast Cancer	Significantly High in Breast Cancer	Specific Subgroups of Breast Cancer ^a^	Reference
Nigeria	12	12	whole blood, hair	whole blood: Cu, Zn, Pb, Se, Cd, Hg, As, Mn, Sr, Ca, Mg, Li, Co hair: Pb, Cd, Hg, As, Cu, Zn, Se, Mn, Sr, Ca, Mg, Li, Co, I, S, P, Ag, Fe, Cr, V, Mo, B, Na, K, Ba, Be, Bi, Ni, Tl, Th, U, Ti, Sn, Al	blood: none hair: Se, Sr, Ca, Co, Na	blood: Cu, Pb, Li hair: Cd, Cu	-	[12]
Italy	80	58	plasma, hair	Cu, Zn, Se	plasma: Se, Zn hair: none	plasma: none hair: none	-	[20]
India	25	25	whole blood	Pb, Cu, Zn, Ca, Fe	Cu	Zn, Fe, Ca	-	[18]
India	160	160	plasma	Se	-	-	-	[21]
Taiwan	25	26	serum	Se, Zn, Cd, Co, Ni, Mn, Fe, Cr, Mg, Al, Ca, Cu, Ag	Se, Zn, Mn, Mg	Cd, Ni, Fe, Cr, Al, Cu	-	[11]
Nigeria	30	30	serum	Cu, Zn, Se	Cu, Zn, Se	-	-	[16]
India	54	54	serum	Ca, Cu, Mg, Fe, P, Zn	Zn	Cu, Fe, P, Ca	-	[15]
China	88	84	serum	Zn, Mn, Al, Cd, Fe, Mg, Ca, Pb, Cu, Se, Ni, Ti, Co, Li, Cr	Mn, Al, Fe, Ti	Cd, Mg, Cu, Co, Li	-	[10]
China	56	20	serum	Cu, Zn, Fe, Se, Mg, Mn	Zn, Se, Mg, Mn	Cu, Fe	-	[14]
Korea	40	144	hair	Ca, Mg, Na, K, Fe, Zn, Cu, Mn, Se, As, P, Cr, U, Cd, Pb, Al, Hg	Ca, Mg, Fe, Zn, Cu, Mn, Pb	Na, K, As	-	[22]
Korea	229	200	serum	Co, Cr, Cu, Mn, Mo, Se, Zn	-	Mn, Mo	-	This study
Malaysia	64	127	hair, toenail	Se	-	-	-	[23]
Malaysia	57	139	hair, toenail	Se	hair: Se toenail: Se	-	-	[24]
Kuwait	50	50	whole blood	Cu, Zn, Se, Cd	Cu, Zn, Se	Cd	Stage I	[17]
Turkey	52	52	hair	Ag, Al, As, Au, B, Ba, Be, Bi, Ca, Cd, Ce, Co, Cr, Cs, Cu, Fe, Ga, Hg, K, Li, Mg, Mn, Na, Ni, Pb, Pd, Rb, Rh, Sb, Sc, Se, Sn, Sr, Ti, V, Zn	Ag, Au, Ba, Be, Bi, Ca, Ga, Hg, Mg, Ni, Pb, Pd, Se, Sn, Sr, Ti, Zn	Al, As, B, Cd, Ce, Co, Cr, Cs, Cu, Fe, K, Li, Mn, Na, Rb, Rh, Sb, V	Stage III	[25]
Turkey	26	27	hair	Cr, Mn	Mn	Cr	Stage III	[26]
Canada	48	96	plasma	Sb, As, Ca, Cd, Cr, Cu, Fe, Mg, Se, Zn	Fe	-	*BRCA1* mutation carriers	[13]
Canada	25	50	toenail	Se	-	-	*BRCA1* mutation carriers	[27]

^a^ Some studies included only specific subtypes of breast cancer.

**Table 4:** Previous studies of trace element concentrations in breast cancer patients compared with controls.

Trace Elements	Region	*n* of Breast Cancer	*n* of Controls	Specimen	Concentration in Breast Cancer	Concentration in Controls	*p*-value	Values	References
Co (µg/L)	Korea	150	137	serum	0.42 (0.18–4.03)	0.31 (0.12–1.21)	<0.0001	mean (range)	This study ^a^
Co (µg/L)	Korea	79	63	serum	0.48 (0.12–2.39)	0.21 (0.12–0.51)	0.1787	mean (range)	This study ^b^
Co (µg/L)	Taiwan	25	26	serum	0.51 ± 0.05	0.49 ± 0.08	NS	mean ± SD	[11]
Co (µg/L)	China ^c^	88	84	serum	2.12 ± 1.33	1.69 ± 0.54	0.006	mean ± SD	[10]
Co (µg/L)	Nigeria	12	12	blood	0.42 (0.2–1.1)	0.53 (0.2–0.9)	0.91	mean (range)	[12]
Cr (µg/L)	Korea	150	137	serum	0.22 (0.12–0.54)	0.21 (0.12–0.51)	0.0767	mean (range)	This study ^a^
Cr (µg/L)	Korea	79	63	serum	0.32 (0.14–4.05)	0.19 (0.10–0.31)	<0.0001	mean (range)	This study ^b^
Cr (µg/L)	Taiwan	25	26	serum	1.36 ± 0.26	0.64 ± 0.20	<0.01	mean ± SD	[11]
Cr (µg/L)	China	88	84	serum	15.0 ± 5.4	13.3 ± 5.4	0.052	mean ± SD	[10]
Cr (µg/L)	Canada	48	96	plasma	3.03 (2.12–4.19)	3.11 (2.12–6.00)	0.35	mean (range)	[13]
Cu (µg/dL)	Korea	150	137	serum	104.18 (65.00–239.00)	96.18 (63.00–149.00)	0.0002	mean (range)	This study ^a^
Cu (µg/dL)	Korea	79	63	serum	90.00 (58.00–128.00)	92.67 (63.00–129.00)	0.2805	mean (range)	This study ^b^
Cu (µg/dL)	Taiwan ^c^	25	26	serum	125.2 ± 15.1	96.5 ± 7.0	<0.01	mean ± SD	[11]
Cu (µg/dL)	Nigeria	30	30	serum	95.3 ± 4.9	65.2 ± 15.0	<0.01	mean ± SD	[16]
Cu (µg/dL)	India	54	54	serum	202.21 ± 89.18	109.56 ± 30.72	<0.001	mean ± SD	[15]
Cu (µg/dL)	China ^c^	88	84	serum	137.2 ± 36.2	113.2 ± 13.6	<0.001	mean ± SD	[10]
Cu (µg/dL)	China ^c^	56	20	serum	115.9 (92.4–14.0)	101.6 (80.5–115.8)	<0.01	mean (range)	[14]
Cu (µg/dL)	Canada ^c^	48	96	plasma	115.4 (36.7–170.6)	113.9 (79.9–237.1)	0.74	mean (range)	[13]
Cu (µg/dL)	Nigeria	12	12	blood	132.5 (105–157)	98.9 (76–145)	<0.001	mean (range)	[12]
Cu (µg/dL)	India ^d^	25	25	blood	65.0 (30.0–150.0)	68.0 (45–130.0)	NS	mean (range)	[18]
Cu (µg/dL)	Kuwait ^e^	50	50	blood	133.0 ± 34.0	147.0 ± 45.0	0.0006	mean ± SD	[17]
Mn (µg/L)	Korea	150	137	serum	1.76 (0.71–2.79)	1.43 (0.61–2.27)	<0.0001	mean (range)	This study ^a^
Mn (µg/L)	Korea	79	63	serum	0.68 (0.37–1.08)	0.64 (0.40–1.66)	0.0099	mean (range)	This study ^b^
Mn (µg/L)	China	88	84	serum	7.4 ± 7.9	19.9 ± 11.0	<0.001	mean ± SD	[10]
Mn (µg/L)	China	56	20	serum	8.30 (5.38–11.75)	10.95 (8.55–12.28)	<0.01	mean (range)	[14]
Mo (µg/L)	Korea	150	137	serum	1.32 (0.59–4.77)	1.10 (0.48–1.99)	0.0039	mean (range)	This study ^a^
Mo (µg/L)Se (µg/L)	Korea	79	63	serum	1.30 (0.70–8.30)	1.08 (0.50–2.20)	0.0056	mean (range)	This study ^b^
	Korea	150	137	serum	106.63 (68.00–179.00)	110.99 (84.00–197.00)	0.0241	mean (range)	This study ^a^
Se (µg/L)	Korea	79	63	serum	100.75 (75.00–154.00)	104.67 (81.00–153.00)	0.0845	mean (range)	This study ^b^
Se (µg/L)	Nigeria	30	30	serum	45.0 ± 4.6	76.4 ± 8.9	<0.01	mean ± SD	[16]
Se (µg/L)	China	88	84	serum	91.4 ± 20.0	95.8 ± 22.7	0.236	mean ± SD	[10]
Se (µg/L)	China	56	20	serum	71.4 (59.2–83.9)	81.3 (72.3–95.3)	<0.01	mean (range)	[14]
Se (µg/L)	Canada	48	96	plasma	100.0 (47.76–1161.2)	85.90 (49.74–933.4)	0.57	mean (range)	[13]
Se (µg/L)	Kuwait	50	50	blood	64.06 ± 16.05	88.64 ± 19.03	<0.0001	mean ± SD	[17]
Se (µg/L)	Iran	45	45	plasma	138.40 ± 40.36	132.15 ± 35.37	NS	mean ± SD	[28]
Zn (µg/dL)	Korea	150	137	serum	96.07 (62.00–150.00)	113.52 (76.00–159.00)	<0.0001	mean (range)	This study ^a^
Zn (µg/dL)	Korea	79	63	serum	77.49 (54.00–111.00)	76.68 (60.00–107.00)	0.5961	mean (range)	This study ^b^
Zn (µg/dL)	Nigeria	30	30	serum	62.7 ± 15.7	93.5 ± 7.2	<0.01	mean ± SD	[16]
Zn (µg/dL)	Pakistan ^c^	65	50	blood	59.7 ± 3.5	100.9 ± 7.5	<0.001	mean ± SD	[29]
Zn (µg/dL)	China ^c^	88	84	serum	111.0 ± 23.6	113.1 ± 21.5	0.824	mean ± SD	[10]
Zn (µg/dL)	China ^c^	56	20	serum	93.9 (77.1–111.0)	106.1 (92.8–119.7)	<0.01	mean (range)	[14]
Zn (µg/dL)	Canada ^c^	48	96	plasma	80.5 (21.9–191.1)	77.2 (57.7–137.9)	0.36	mean (range)	[13]
Zn (µg/dL)	Kuwait ^e^	50	50	blood	99.0 ± 39.0	360 ± 110.0	<0.0001	mean ± SD	[17]

Abbreviations: SD, standard deviation; NS, not significant. ^a^ Results of the discovery cohort in this study; ^b^ results of the validation cohort in this study; ^c^ original results were converted from µg/L to µg/dL for comparison; ^d^ original results were converted from µg/mL to µg/dL for comparison; ^e^ original results were converted from mg/L to µg/dL for comparison.

## Supplementary Materials

The following are available online at http://www.mdpi.com/2072-6643/11/1/37/s1, Material S1: Details in sample preparation and analytical methods, Table S1: Trace element levels in Korean female control subjects, Table S2: Trace element levels in Korean female breast cancer patients, Table S3: Correlation among trace element levels, Figure S1: Overall study design and number of participants included in the discovery and validation cohorts. Serum levels of seven trace elements in breast cancer patients and controls. In the discovery cohort, subjects with benign breast disease were not included in the control population. In the validation cohort, 44 female subjects with benign breast disease were included in the control population. Dashed lines represent subgroup analysis according to further information about the presence of lymph node metastasis, distant metastasis (stage IV breast cancer), and triple-negative breast cancer. Those with triple-negative cancer tested negative for all three of the following markers on post-surgical information: estrogen receptor (ER), progesterone receptor (PR), and human epidermal growth factor receptor 2 (HER2). Among 79 breast cancer patients, none had stage IV breast cancer and thus subgroup analysis could not be performed.
