# Supplementary material for: Serum Trace Elements and Their Associations with Breast Cancer Subgroups in Korean Breast Cancer Patients
Source: Nutrients. 2018 Dec 24;11(1):37. doi: 10.3390/nu11010037 (PMC6357144; doi:10.3390/nu11010037)
Supplement: Supplementary file 1 [file nutrients-11-00037-s001.zip › nutrients-390165-supplementary/1. Material S1_.docx]

**Serum Trace Elements and Their Associations with Breast Cancer Subgroups in Korean Breast Cancer Patients**

Material S1. Details of sample preparation and analytical methods for ICP-MS

Sample Preparation

The following chemicals were used to prepare collected serum samples, standards, and control materials: nitric acid (65%, JKC, Chungcheongnam-do, Korea), ammonium hydroxide solution (28% NH_3_ in H_2_O, Sigma-Aldrich, Schnelldorf, Germany), Triton X100 (t-octylphenoxypolyethoxyethanol, BioXtra, Sigma-Aldrich), 1-butanol (anhydrous, 99.8%, Sigma-Aldrich) and EDTA (H4-EDTA, 99.995%, Sigma-Aldrich). We used multi-element standards (environmental calibration standard 5183-4688, Agilent, Santa Clara, CA, USA) for Cr, Co, Mn, and Mo with additional single-element standards for Cu (1000 ppm, Wako, Osaka, Japan), Se (1000 ppm, SCP SCIENCE, Quebec, Canada), and Zn (1000 ppm, Wako), traceable according to the National Metrology Standard for preparation. Internal standard solutions of Ge (1000 ppm, Wako) for Se, In (1000 ppm, Perkin Elmer, Shelton, USA) for Mo and Mn, Rh (1000 ppm, Wako) for Cu and Zn, and Sc (1000 ppm, PerkinElmer) for Cr and Co were used. For dilutions, aliquots (0.2 mL) of the serum samples were diluted with 2 mL of dilution solution consisting of 1.5% (w/v) 1-butanol, 0.05% (w/v) EDTA, 0.05% (w/v) triton X-100, 0.14% (w/v) NH_4_OH and installed on a vortex mixer (Vortex Genie 2, Scientific Industries, NY, USA) before ICP-MS analysis. The blanks, standards, and control materials were prepared in the same manner as the samples.

Operating Conditions of ICP-MS

Details of the operating conditions of ICP-MS are summarized in the following tables (Material S1 Tables 1).

**Material S1 Table 1**. Typical operating parameters for ICP-MS (Agilent 7900)

| Agilent 7900 ICP-MS  Plasma RF power (W) | Operating conditions  Forward power 1550  Reflected power 2 |
| --- | --- |
| Sampling depth (mm) | 8.0 |
| Plasma gas flow (L/min) | 15 |
| Carrier gas flow (L/min) | 1.00 |
| Makeup gas flow (L/min) | 0.1 |
| Sample uptake rate (mL/min) | 0.5 (with nebulizer pump speed of 0.1 rps, tubing of 1.02 mm i.d. for sample)  Stabilization: 30 sec |
| Spray chamber cooling temperature (°C) | 2 |
| Sampling cone | Ni, 1 mm diameter orifice |
| Skimmer cone | Ni, 0.45 mm diameter orifice |
| Cell gas mode | He (3 mL/min for Co, Cu, Mo, Mn, and Se, 5 mL/min for Cr, and 6 mL/min for Zn) |
| Isotopes | ^53^Cr, ^55^Mn, ^59^Co, ^63^Cu, ^66^Zn, ^82^Se, ^95^Mo |
| Internal standards | ^45^Sc, ^72^Ge, ^103^Rh, ^115^In |
| Replicates | 3 |
| Sweeps per replicate | 100 |
