# Supplementary material for: Serum Trace Elements and Their Associations with Breast Cancer Subgroups in Korean Breast Cancer Patients
Source: Nutrients. 2018 Dec 24;11(1):37. doi: 10.3390/nu11010037 (PMC6357144; doi:10.3390/nu11010037)
Supplement: Supplementary file 1 [file nutrients-11-00037-s001.zip › nutrients-390165-supplementary/2. Supplementary Tables.docx]

**Table S1.** Trace element levels in Korean female control subjects.

|  | 1st dataset for discovery cohort | | | | | | | | |  | 2nd dataset for validation cohort | | | | | | | | |
| --- | --- | --- | --- | --- | --- | --- | --- | --- | --- | --- | --- | --- | --- | --- | --- | --- | --- | --- | --- |
|  | n | Mean | SD | Min | 1st quartile | Med | 3rd quartile | Max | *p*-value^a^ |  | n | Mean | SD | Min | 1st quartile | Med | 3rd quartile | Max | *p*-value^a^ |
| Age (years) | 137 | 48.59 | 11.05 | 26.00 | 42.75 | 48.00 | 56.00 | 73.00 | 0.06 |  | 63 | 40.73 | 10.91 | 21.00 | 31.00 | 41.00 | 48.00 | 73.00 | 0.22 |
| Body mass index (kg/m^2^) | 137 | 21.83 | 3.00 | 16.20 | 19.80 | 21.40 | 23.80 | 34.00 | <0.01 |  | 63 | 21.58 | 2.35 | 16.80 | 19.86 | 21.45 | 23.23 | 27.43 | 0.17 |
| Serum total protein (g/dL) | 137 | 7.10 | 0.43 | 5.70 | 6.80 | 7.10 | 7.40 | 8.60 | 0.15 |  | 63 | 7.26 | 0.41 | 6.30 | 7.00 | 7.30 | 7.50 | 8.20 | 0.29 |
| Serum albumin (g/dL) | 137 | 4.37 | 0.32 | 3.50 | 4.20 | 4.30 | 4.60 | 5.40 | <0.01 |  | 63 | 4.60 | 0.23 | 4.10 | 4.50 | 4.60 | 4.78 | 5.10 | 0.17 |
| Serum total cholesterol (mg/dL) | 137 | 195.18 | 31.46 | 136.00 | 172.00 | 193.00 | 218.00 | 298.00 | 0.09 |  | 63 | 175.97 | 30.53 | 111.00 | 157.50 | 169.00 | 192.50 | 263.00 | 0.04 |
| Aspartate transaminase (IU/L) | 137 | 21.43 | 7.83 | 10.00 | 17.00 | 20.00 | 24.00 | 71.00 | <0.01 |  | 63 | 18.51 | 6.14 | 11.00 | 15.00 | 17.00 | 21.00 | 53.00 | <0.01 |
| Alanine transaminase (IU/L) | 137 | 17.75 | 10.47 | 4.00 | 11.00 | 15.00 | 20.25 | 69.00 | <0.01 |  | 63 | 14.98 | 8.25 | 5.00 | 10.00 | 12.00 | 17.75 | 53.00 | <0.01 |
| Alkaline phosphatase (U/L) | 137 | 58.56 | 24.11 | 21.00 | 44.00 | 54.00 | 67.00 | 230.00 | <0.01 |  | 63 | 56.44 | 19.26 | 20.00 | 43.00 | 52.00 | 67.75 | 140.00 | <0.01 |
| High-density lipoproteins (mg/dL) | 129 | 63.22 | 15.83 | 24.00 | 53.00 | 62.00 | 72.25 | 134.00 | <0.01 |  | 42 | 69.64 | 14.75 | 41.00 | 60.00 | 68.50 | 78.00 | 107.00 | 0.57 |
| Low-density lipoprotein (mg/dL) | 130 | 119.24 | 29.38 | 38.00 | 98.00 | 117.00 | 138.00 | 190.00 | 0.10 |  | 42 | 111.21 | 31.96 | 55.00 | 93.00 | 102.50 | 124.00 | 208.00 | <0.01 |
| Cobalt (µg/L) | 137 | 0.31 | 0.20 | 0.00 | 0.18 | 0.24 | 0.36 | 1.21 | <0.01 |  | 63 | 0.48 | 0.21 | 0.15 | 3.23 | 0.45 | 0.59 | 1.11 | 0.02 |
| Chromium (µg/L) | 137 | 0.21 | 0.06 | 0.12 | 0.18 | 0.20 | 0.23 | 0.51 | <0.01 |  | 63 | 0.19 | 0.05 | 0.10 | 0.15 | 0.18 | 0.23 | 0.31 | 0.09 |
| Copper (µg/dL) | 137 | 96.18 | 13.33 | 63.00 | 87.00 | 95.00 | 103.00 | 149.00 | <0.01 |  | 63 | 92.67 | 15.20 | 63.00 | 81.25 | 92.00 | 102.50 | 129.00 | 0.63 |
| Manganese (µg/L) | 137 | 1.43 | 0.42 | 0.61 | 1.04 | 1.54 | 1.71 | 2.27 | <0.01 |  | 63 | 0.64 | 0.22 | 0.40 | 0.53 | 0.58 | 0.68 | 1.66 | <0.01 |
| Molybdenum (µg/L) | 137 | 1.10 | 0.29 | 0.48 | 0.88 | 1.05 | 1.30 | 1.99 | 0.04 |  | 63 | 1.08 | 0.36 | 0.50 | 0.90 | 1.10 | 1.20 | 2.20 | <0.01 |
| Selenium (µg/L) | 137 | 110.99 | 15.19 | 84.00 | 102.00 | 109.00 | 119.00 | 197.00 | <0.01 |  | 63 | 104.67 | 13.94 | 81.00 | 95.00 | 102.00 | 112.00 | 153.00 | <0.01 |
| Zinc (µg/dL) | 137 | 113.52 | 19.61 | 76.00 | 97.75 | 110.00 | 126.00 | 159.00 | <0.01 |  | 63 | 76.68 | 11.29 | 60.00 | 67.00 | 75.00 | 85.00 | 107.00 | 0.04 |

Abbreviations: Max, maximum; Med, median; Min, minimum; SD, Standard deviation

^a^*p*-value based on the Shapiro-Wilks test for normality

**Table S2.** Trace element levels in Korean female breast cancer patients.

|  | 1st dataset for discovery cohort | | | | | | | | |  | 2nd dataset for validation cohort | | | | | | | | |
| --- | --- | --- | --- | --- | --- | --- | --- | --- | --- | --- | --- | --- | --- | --- | --- | --- | --- | --- | --- |
|  | n | Mean | SD | Min | 1st quartile | Med | 3rd quartile | Max | *p*-value^a^ |  | n | Mean | SD | Min | 1st quartile | Med | 3rd quartile | Max | *p*-value^a^ |
| Age (years) | 150 | 47.07 | 10.68 | 27.00 | 40.00 | 46.00 | 53.00 | 78.00 | 0.01 |  | 79 | 48.60 | 8.79 | 28.00 | 43.00 | 47.00 | 53.75 | 78.00 | 0.16 |
| Body mass index (kg/m^2^) | 150 | 23.12 | 3.25 | 16.64 | 20.80 | 22.54 | 24.53 | 32.44 | <0.01 |  | 79 | 23.06 | 2.70 | 17.77 | 21.32 | 23.11 | 24.82 | 29.72 | 0.62 |
| Serum total protein (g/dL) | 150 | 7.30 | 0.41 | 6.00 | 7.10 | 7.30 | 7.60 | 8.30 | 0.27 |  | 79 | 7.44 | 0.44 | 6.70 | 7.10 | 7.40 | 7.70 | 8.50 | 0.02 |
| Serum albumin (g/dL) | 150 | 4.62 | 0.30 | 2.90 | 4.50 | 4.70 | 4.80 | 5.20 | <0.01 |  | 79 | 4.61 | 0.24 | 3.70 | 4.50 | 4.60 | 4.80 | 5.10 | <0.01 |
| Serum total cholesterol (mg/dL) | 150 | 187.45 | 37.04 | 107.00 | 163.00 | 184.50 | 210.00 | 360.00 | <0.01 |  | 79 | 182.05 | 31.36 | 113.00 | 163.00 | 177.00 | 202.75 | 303.00 | 0.02 |
| Aspartate transaminase (IU/L) | 150 | 21.39 | 19.65 | 9.00 | 15.00 | 18.00 | 21.00 | 237.00 | <0.01 |  | 79 | 18.76 | 6.62 | 8.00 | 15.00 | 17.00 | 21.00 | 51.00 | <0.01 |
| Alanine transaminase (IU/L) | 150 | 17.61 | 11.16 | 5.00 | 11.00 | 14.00 | 21.00 | 78.00 | <0.01 |  | 79 | 15.96 | 6.74 | 6.00 | 12.00 | 14.00 | 18.00 | 39.00 | <0.01 |
| Alkaline phosphatase (U/L) | 149 | 62.94 | 28.24 | 7.00 | 47.00 | 57.00 | 72.00 | 249.00 | <0.01 |  | 79 | 57.48 | 16.94 | 32.00 | 45.25 | 53.00 | 64.00 | 108.00 | <0.01 |
| High-density lipoproteins (mg/dL) | 75 | 60.23 | 14.02 | 35.00 | 50.00 | 58.00 | 67.00 | 107.00 | <0.01 |  | 72 | 62.61 | 15.29 | 31.00 | 51.00 | 62.50 | 73.00 | 97.00 | 0.54 |
| Low-density lipoprotein (mg/dL) | 75 | 118.69 | 30.18 | 62.00 | 95.50 | 115.00 | 135.75 | 207.00 | 0.04 |  | 72 | 115.63 | 30.57 | 41.00 | 95.00 | 109.00 | 135.00 | 219.00 | 0.04 |
| Cobalt (µg/L) | 150 | 0.42 | 0.37 | 0.18 | 0.25 | 0.31 | 0.43 | 4.03 | <0.01 |  | 79 | 0.48 | 0.36 | 0.12 | 0.25 | 0.41 | 0.56 | 2.39 | <0.01 |
| Chromium (µg/L) | 150 | 0.22 | 0.06 | 0.12 | 0.18 | 0.21 | 0.24 | 0.54 | <0.01 |  | 79 | 0.32 | 0.44 | 0.14 | 0.20 | 0.27 | 0.31 | 4.05 | <0.01 |
| Copper (µg/dL) | 150 | 104.18 | 21.57 | 65.00 | 93.00 | 100.00 | 111.00 | 239.00 | <0.01 |  | 79 | 90.00 | 14.05 | 58.00 | 82.00 | 90.00 | 96.75 | 128.00 | 0.07 |
| Manganese (µg/L) | 150 | 1.76 | 0.38 | 0.71 | 1.49 | 1.75 | 2.00 | 2.79 | 0.16 |  | 79 | 0.68 | 0.16 | 0.37 | 0.56 | 0.64 | 0.78 | 1.08 | 0.01 |
| Molybdenum (µg/L) | 150 | 1.32 | 0.62 | 0.59 | 0.95 | 1.16 | 1.50 | 4.77 | <0.01 |  | 79 | 1.30 | 0.86 | 0.70 | 0.93 | 1.20 | 1.40 | 8.30 | <0.01 |
| Selenium (µg/L) | 150 | 106.63 | 15.76 | 68.00 | 98.00 | 107.00 | 114.00 | 179.00 | <0.01 |  | 79 | 100.75 | 14.06 | 75.00 | 92.00 | 98.00 | 112.50 | 154.00 | <0.01 |
| Zinc (µg/dL) | 150 | 96.07 | 14.36 | 62.00 | 87.00 | 95.00 | 104.00 | 150.00 | 0.09 |  | 79 | 77.49 | 10.45 | 54.00 | 72.00 | 76.00 | 82.75 | 111.00 | 0.03 |

Abbreviations: Max, maximum; Med, median; Min, minimum; SD, Standard deviation

^a^*p*-value based on the Shapiro-Wilks test for normality

**Table S3.** Correlation among trace element levels.

|  |  | 1st dataset for discovery cohort | |  | 2nd dataset for validation cohort | |
| --- | --- | --- | --- | --- | --- | --- |
|  |  | Correlation coefficient | *p*-value |  | Correlation coefficient | *p*-value |
| Cobalt | Chromium | 0.0850 | 0.1507 |  | 0.1310 | 0.1196 |
|  | Copper | -0.0502 | 0.3968 |  | -0.0360 | 0.6691 |
|  | Manganese | 0.1197 | 0.0427^a^ |  | 0.2210 | 0.0081^a^ |
|  | Molybdenum | -0.0357 | 0.5470 |  | 0.0890 | 0.2909 |
|  | Selenium | -0.1415 | 0.0164^a^ |  | -0.0360 | 0.6686 |
|  | Zinc | -0.1396 | 0.0179^a^ |  | 0.0810 | 0.3381 |
| Chromium | Copper | 0.1462 | 0.0132^a^ |  | -0.1230 | 0.1434 |
|  | Manganese | 0.0131 | 0.8245 |  | 0.1560 | 0.0634 |
|  | Molybdenum | 0.0164 | 0.7821 |  | 0.8470 | <0.0001 ^a^ |
|  | Selenium | 0.0861 | 0.1458 |  | -0.1270 | 0.1332 |
|  | Zinc | -0.0539 | 0.3631 |  | -0.0310 | 0.7152 |
| Copper | Manganese | 0.0653 | 0.2704 |  | -0.0360 | 0.6677 |
|  | Molybdenum | 0.0928 | 0.1168 |  | -0.0810 | 0.3409 |
|  | Selenium | 0.2216 | 0.0002^a^ |  | 0.3860 | <0.0001^a^ |
|  | Zinc | -0.1046 | 0.0770 |  | 0.1660 | 0.0480^a^ |
| Manganese | Molybdenum | 0.0442 | 0.4561 |  | 0.0660 | 0.4342 |
|  | Selenium | 0.0495 | 0.4039 |  | -0.0220 | 0.7921 |
|  | Zinc | 0.0984 | 0.0963 |  | 0.2040 | 0.0147^a^ |
| Molybdenum | Selenium | -0.0976 | 0.0989 |  | -0.0950 | 0.2622 |
|  | Zinc | -0.0820 | 0.1661 |  | -0.1040 | 0.2201 |
| Selenium | Zinc | 0.1136 | 0.0545 |  | 0.1820 | 0.0304^a^ |

^a^*p* < 0.05

Weak positive correlations between cobalt and manganese, and selenium and copper were observed for both cohorts in common.
