# Supplementary figures and images for: Serum Trace Elements and Their Associations with Breast Cancer Subgroups in Korean Breast Cancer Patients
Source: Nutrients. 2018 Dec 24;11(1):37. doi: 10.3390/nu11010037 (PMC6357144; doi:10.3390/nu11010037)

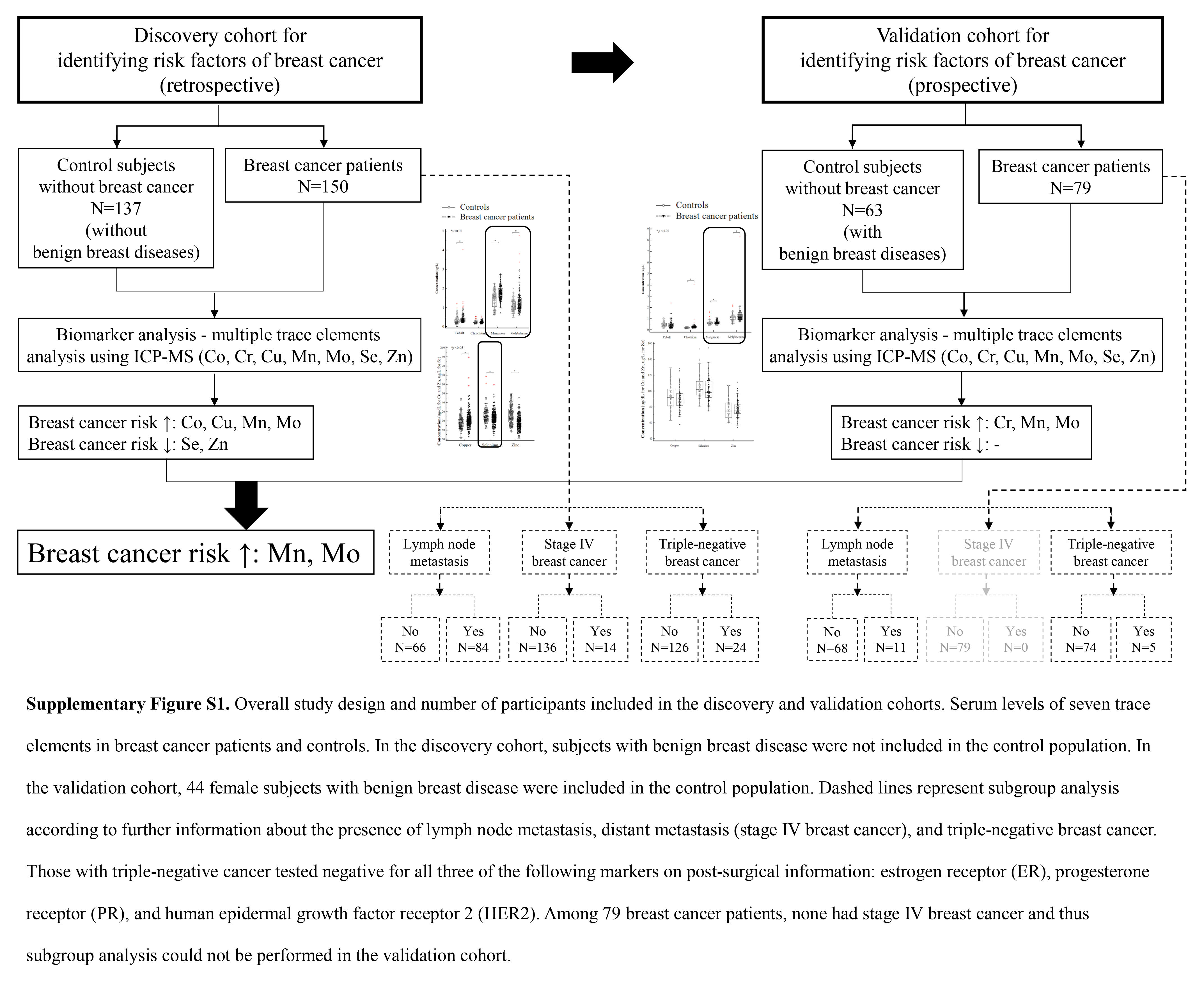

Supplement: Supplementary file 1 [file nutrients-11-00037-s001.zip › nutrients-390165-supplementary/3. Supplementary Figure S1.tif]
